# CXCR5, the Defining Marker for Follicular B Helper T (T_FH_) Cells

**DOI:** 10.3389/fimmu.2015.00296

**Published:** 2015-06-08

**Authors:** Bernhard Moser

**Affiliations:** ^1^Institute of Infection and Immunity, Cardiff University, Cardiff, UK

**Keywords:** CXCR5, chemokine receptor, T_FH_ cell, migration, humoral immune response

The discovery of follicular B helper T (T_FH_) cells has its roots in the early 90s, the “childhood” of chemokine research that has since grown into an independent, global specialty within immunology. The class of chemoattractant proteins with shared structural features was named “chemokines,” and early work with non-chemokine (FMLP, C5a) and chemokine (IL-8/CXCL8) receptors revealed that chemokine receptors belong to the large family of G-protein-coupled receptors (GPCRs) distinguished by their prototypical seven-transmembrane protein architecture. The search for novel chemokines and their receptors greatly intensified during that time, because it became increasingly clear that this novel cytokine system is essential for controlling immune cell mobilization and tissue localization and, hence, for controlling the entirety of immune processes in health and disease. Molecular identification of chemokine receptors led to the identification of immune cells that responded to the corresponding chemokine ligands and allowed their tracking during acute and chronic immune responses. At the last count, the inventory of chemokine receptors that, together with adhesion receptors, make up the address codes on human immune cells includes 18 individual members recognizing one or multiple of a total of 45 chemokines. Further underscoring the complexity of the chemokine system, we also know of six atypical chemokine receptors, some of which control chemokine positioning and degradation.

Since my earliest steps in research, I was fascinated by the cytokine network controlling the highly complex interactions between immune cells and their functions in immune defense. In fact, an ambitious project aimed at the molecular characterization of the elusive “antigen-specific T helper factors” tipped the balance in favor of carrying out my Ph.D. studies in the lab of Profs. D. G. Kilburn, R. C. Miller Jr., and R. A. J. Warren at the University of British Columbia in Vancouver. It was not the skiing in the Whistler Mountains nor the salmon fishing along the Sunshine Coast that did the job, as suggested by some of my colleagues at the Federal Institute of Technology in Zurich. Needless to say that the molecular identification of the T cell antigen receptor in 1984 brought our project to an immediate halt. Still, when the question about postdoc projects arose, I was fascinated by the new world of chemotactic cytokines (to be called “chemokines” a few years later) that I was introduced to by Prof. Marco Baggiolini, the director at the Theodor-Kocher Institute of the University of Bern. Therefore, upon arrival at the Theodor-Kocher Institute in 1989, I was determined to clone the receptor for NAF, the first chemokine with selectivity for neutrophils (now known as IL-8 or CXCL8 according to the systematic chemokine nomenclature). This young field of research turned out to be highly competitive, not least because of its translational potential. Unsurprisingly, we were beaten by two labs who reported the cloning of the CXCL8 receptors well before our own initiative had a chance to take off (1, 2). As a small consolation, we succeeded to be first in demonstrating that human neutrophils carried two types of CXCL8 receptors on their cell surface distinguished by their variable affinity for other CXCL8-related chemokines (3, 4). Still, our multipronged cloning efforts paid off and revealed numerous orphan GPCRs with similarity to the CXCL8 receptors. In a great team effort by many colleagues, including Marcel Loetscher, Daniel Legler, Patrick Schaerli, and Regula Stuber-Roos, together with the protein chemist Ian Clark-Lewis at the Biomedical Research Centre of the University of British Columbia (who sadly died in 2002), we were then able to “deorphanize” some of these novel GPCRs in the subsequent years.

As part of our chemokine receptor cloning initiatives, Luca Barella, who was a Ph.D. student in my lab, identified and characterized an orphan GPCR, termed monocyte-derived receptor 15 (MDR15) (5), which turned out to be a structural variant of Burkitt’s lymphoma receptor 1 (BLR1) published several years ahead of us by Martin Lipp’s group in Berlin (6). In fact, we first heard about BLR1 during a conversation with Martin Lipp whom we met in July 1992 at the fifth International Congress on Cell Biology in Madrid. Based on the structural similarities to the chemokine receptors that were known at that time (CXCR1, CXCR2, CCR1, and CCR2), it was clear to us that MDR15/BLR1 must be a novel chemokine receptor. However, none of the known chemokines bound to it.

Intriguingly, MDR15/BLR1 transcripts were primarily found to be present in the lymphocyte fraction of peripheral blood mononuclear cells, and most notably in chronic B leukemia cell lines, but not in cells characterized by the other known chemokine receptors. It took another 3 years to “deorphanize” MDR15/BLR1. While searching expressed sequence tag (EST) cDNA databanks, Daniel Legler, a Ph.D. student at that time, identified a novel chemokine, which we termed B cell-attracting chemokine 1 (BCA-1; now officially known as CXCL13) because of its efficacious chemoattractant activity for B cells (7). The mouse ortholog of BCA-1/CXCL13 was published by the group of Michael Gunn at UCSF within the same month (8). Importantly, Michael Gunn and our group found that BCA-1/CXCL13 was the selective chemokine ligand for mouse and human MDR15/BLR1 (now officially known as CXCR5), respectively. Of note, the highly selective expression of CXCL13 in secondary lymphoid tissues matched perfectly well the findings of Martin Lipp’s group about the importance of BLR1 in the localization of B cells within murine secondary lymphoid tissues (9).

In addition to B cells, we noticed that a large fraction of CD4^+^ memory T cells present in tonsils expressed CXCR5. By contrast, CXCR5^+^ T cells were relatively scarce in peripheral blood of healthy individuals. We also found that its single ligand CXCL13 was discretely expressed in the follicular mantle zone but not in the paracortical T cell zone or high endothelial venules where the CCR7-specific chemokines CCL19 and CCL21 are present. Unlike CCL19/CCL21, it appeared that CXCL13 did not play a role in the recruitment of T cells (and B cells) into lymph nodes but instead controlled the segregation of lymphocytes between T cell and B cell compartments, as Michael Gunn’s group has shown for BLC in mice (10). Could it be that tonsillar CXCR5^+^ T cells corresponded to the elusive T helper cell subset postulated to control B cell responses to protein antigens? Indeed, tonsillar B cells produced large amounts of isotype-switched antibodies during co-culture with CXCR5^+^ T cells but not CXCR5^-^ T cells. During a subsequent discussion with Martin Lipp, who contributed his CCR7-specific antibodies to our study, we found out that both of our groups had similar results and, therefore, we agreed to submit our findings as back-to-back manuscripts to the *Journal of Experimental Medicine* (11, 12). Together with the journal editors, we then decided to designate this novel T helper subset as T_FH_ cells, which today is also known as “follicular helper T cells” and “B helper T cells” (Figure 1).

**Figure 1:**
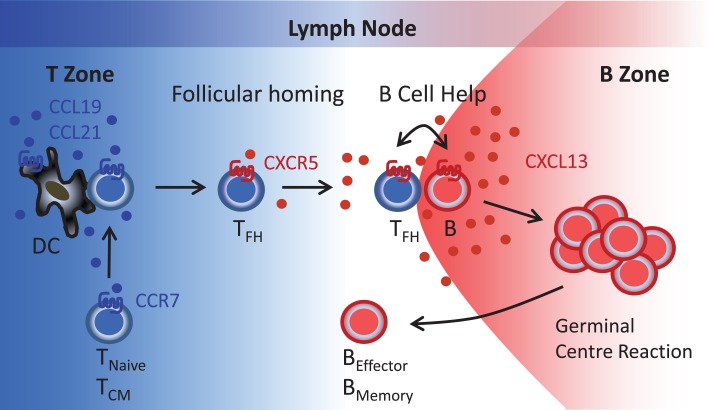
**Follicular B helper T (T_FH_) cells**. T cells reach the lymph node paracortical region (T zone) *via* high endothelial venules (blood) or afferent lymphatics (peripheral tissues) in response to the CCR7-specific chemokines CCL19 and CCL21. During contact with antigen-presenting DC, T cells become primed that includes induction of CXCR5 expression. Reduced CCR7 expression allows newly generated CXCR5^+^ T (T_FH_) cells to respond to follicular CXCL13 and to relocate to the B cell follicles where T_FH_-B cell interactions involving a series of co-stimulatory receptors (CD40, ICOS, etc.) and cytokines (IL-10, IL-21, etc.) initiate the germinal center reaction and the formation of antibody-secreting plasma cells and memory B cells. T_Naive_, naïve T cells; T_CM_, central memory T cells; T_FH_, follicular B helper T cells; B, B cells; B_Effector_, plasma cells; B_Memory_, memory B cells.

The separation of T helper cells into Th1 and Th2 cells was instrumental in delineating immune responses to distinct classes of pathogens, such as viruses and intracellular bacteria for Th1 cells and extracellular pathogens and allergens for Th2 cells.

Following the fundamental dogma underscoring the inseparable relationship between tissue localization and immune cell function, we and Martin Lipp’s group jointly discovered T_FH_ cells as a third distinct T helper cell subset, which was highlighted in a commentary by Charles Mackay (13). An avalanche of murine studies by numerous outstanding labs worldwide confirmed and extended our initial findings about the role played by T_FH_ cells in humoral immunity. Thanks to their efforts, it is now clear that defects in their generation and/or function have a profound effect on antibody dependent immune responses. In fact, increased numbers of T_FH_ cells are now known to be associated with B cell autoimmunity and lymphomas whereas defects in T_FH_ cell generation cause severe humoral immunodeficiency [reviewed in Ref. (14)]. Finally, since the presence of CXCR5^+^ T cells in peripheral blood reflects ongoing humoral immune responses (15), CXCR5 may even serve as a unique and convenient biomarker for the evaluation of ongoing vaccination responses. Today, T_FH_ cells have a firm place among an increasing number of T helper cell subsets distinguished by their characteristic migration and functional properties.

## Conflict of Interest Statement

The author declares that the research was conducted in the absence of any commercial or financial relationships that could be construed as a potential conflict of interest.
